# The effect of eplerenone on the renin‐angiotensin‐aldosterone system of rats with thyroid dysfunction

**DOI:** 10.1111/jphp.13168

**Published:** 2019-10-03

**Authors:** Kawa Dizaye, Zana A. Mustafa

**Affiliations:** ^1^ College of Medicine Hawler Medical University Erbil Iraq; ^2^ Department of Pharmacy Medical Technical Institute Erbil Polytechnic University Erbil Iraq

**Keywords:** aldosterone, angiotensin, eplerenone, hyperthyroidism, hypothyroidism, renin

## Abstract

**Objectives:**

This study was conducted to evaluate the effect of eplerenone on the RAAS and kidney function in rats with thyroid hormone disorders.

**Methods:**

This study involved 30 male Wistar albino rats, divided into three groups. The first group (*N* = 6) served as a control. The second group involved 12 rats with experimentally induced hypothyroidism through receiving propylthiouracil (0.05% w/v) in drinking water for one month, which was divided into two subgroups of six rats each. The first subgroup served as a positive hypothyroid control, and the second subgroup received oral daily dose of eplerenone (100 mg/kg) for 14 days. The third group included 12 rats with induced hyperthyroidism with L‐thyroxin (0.0012% w/v) in drinking water, and rats in this group were also divided into two subgroups. The first subgroup served as a positive hyperthyroid control, and the second subgroup received oral eplerenone 100 mg/kg.

**Results:**

Eplerenone indicated a significant increase in renin and angiotensin I from 184.09 pg/ml and 178.66 pg/ml to 603.31 pg/ml and 250.88 pg/ml, respectively, meanwhile, aldosterone indicated no significant changes after inducing hypothyroidism and eplerenone administration.

The induction of hyperthyroidism led to a significant increase in angiotensin I from 248.84 pg/ml to 292.22 pg/ml. Oral administration of eplerenone for 14 days caused a significant increase aldosterone from 364.23 pg/ml to 497.02 pg/ml.

**Conclusion:**

Eplerenone significantly increased the serum renin and angiotensin I in hypothyroid and aldosterone and angiotensin I in hyperthyroid rats. Aldosterone in hypothyroid rats was not changed by eplerenone.

**Abbreviations**
RAAS: Renin‐angiotensin‐aldosterone system
ACE: Angiotensin converting enzyme
ANG II: Angiotensin II
TSH: Thyroid stimulating hormone
ELISA: Enzyme‐linked immunosorbent assay
GFR: Glomerular filtration rate

## Introduction

The renin‐angiotensin‐aldosterone system (RAAS) plays a major role in maintaining hemodynamic stability in response to the decreased blood volume, water and salts.^1^, ^2^ It mainly acts on the kidneys, but it also on the brain, heart, blood vessels and adrenal glands.^3^

Recent studies have suggested that AT_2_R‐mediated signalling induces inhibition of cell growth or apoptosis by counteracting the AT_1_R signal.^4^ The basal systolic blood pressure of AT_2_R‐null mice is mildly elevated compared with wild‐type (wild) mice.^5^ However, the AT_2_R is thought to be expressed in glomeruli in response to RAAS activation, such as during sodium depletion.^6^ The mechanism of AT_2_R signalling has not yet been fully elucidated.

The mineralocorticoid aldosterone is the chain end of the RAAS that regulates water–sodium reabsorption and potassium excretion in late distal tubules and collecting duct. It influences blood volume and blood pressure. The aldosterone level in the blood is affected by many factors such as angiotensin II, potassium level^,^ atrial natriuretic peptide, vasopressin, adrenocorticotropic hormone and vasoactive intestinal peptide.^7^, ^8^, ^9^

Extreme increases of aldosterone in the blood may produce a number of pathophysiological effects such as hypertension, sodium retention and fibrosis. The mentioned negative effects of aldosterone have been indicated to be remedied by mineralocorticoid receptor antagonists.^10^, ^11^, ^12^, ^13^

Eplerenone is an aldosterone receptor antagonist that has been introduced for the management of hypertention,^14^, ^15^ also proven to be effective in reducing cardiovascular diseases secondary to hypertension,^16^, ^17^ this drug has also been shown to reduce mortality in patients with heart failure after myocardial infarction.^18^, ^19^

When compared to the older aldosterone receptor blocking agent spironolactone, Eplerenone has higher affinity for aldosterone receptor and much less affinity for progesterone receptors (<1%) and androgen receptors (0.1%).^20^ Recent animal trials have shown that eplerenone reduces the extent of necrosis in myocardial infarction also reduces arteriopathy in hypertensive and heart failure patients.^21^

Thyroid hormone is one of the main regulators of metabolic processes important for growth, development and metabolism in adults.^22^, ^23^, ^24^ Stimulation of the gland by thyroid‐stimulating hormone (TSH) triggers the secretion of both triiodothyronine and thyroxine into the blood. It is well established that the thyroid hormone level is associated with body weight and energy consumption.^25^, ^26^, ^27^ Hyperthyroidism is characterized by increased metabolic rate, weight loss, energy expenditure, reduced circulating cholesterol, enhanced lipid breakdown and gluconeogenesis.^28^, ^29^ By comparison, hypothyroidism, which is reduced thyroid hormone release, is correlated with reduced metabolic rate, reduced energy consumption, weight gain, increased cholesterol levels, lipid breakdown and reduced gluconeogenesis.^30^

Thyroid hormones play a critical role in blood pressure control and have many effects on the cardiovascular system.^31^, ^32^, ^33^, ^34^ One of the main physiological actions of thyroid hormones is increasing tissue response to the effects of the sympathetic system, and it is considered one of the main determinants of regulating blood pressure. Furthermore, it is known that thyroid hormones might stimulate the RAAS by a different mechanism without the involvement of the sympathetic system.^35^

Based on the results of some studies, thyroid hormones could also affect renin production and its release by the cells of the juxtaglomerular apparatus and serum renin activity.^36^, ^37^ Increased sympathetic outflow was thought to be a major cause of increased plasma renin activity in hyperthyroidism;^38^ however, increased sympathetic outflow has been observed in hypothyroidism.^39^ Adding to that, the removal of the thyroid gland 36 and methimazole‐induced hypothyroidism reduces serum renin activity.^37^

According to our knowledge, no data are available about the effect of thyroid level on the efficacy of eplerenone. We hypothesized that thyroid gland disorder could affect eplerenone’s efficacy that will consequently be affecting RAAS axis, renal and blood pressure. For this purpose, effect of eplerenone on the renin‐angiotensin‐aldosterone system and kidney function in rats with thyroid hormone disorders was evaluated, Eplerenone was chosen because thyroid problems are a common disease in our region and eplerenone is widely prescribed for the treatment of heart failure and hypertension as it has lower antiandrogenic effects compared with spironolactone.

## Materials and Methods

### Animals

Thirty male Wistar albino rats, weighing between 300 and 400 g, were used in this study. The animals were purchased from the Biology Department, College of Education, University of Salahaddin. The rats were kept in the animal house of the College of Medicine, Hawler Medical University, at room temperature (with cycles of 12 h light and 12 h dark). The rats were fed with standard rat food and had free access to water.

### Ethics committee approval

This work was approved by the ethics committee of the College of Medicine, Hawler Medical University, with the code number 7 on 15‐1‐2018.

### Materials

Serum renin, angiotensin I, angiotensin II, aldosterone and TSH rat kits for enzyme‐linked immunosorbent assay (ELISA) were purchased from Elabscience (Houston, texas, USA). A Coda monitor noninvasive blood pressure system from Kent Scientific was used to measure blood pressure and heart rate. L‐thyroxine 100 µg tablet (Merck pharmaceurticals, Kenilworth, New Jersey, USA), eplerenone 25 mg tablet (Pfizer, New York City, USA) tablet and propylthiouracil 50 mg tablet (Actavis, Devonshire, UK) tablet were purchased from a pharmacy.

### Study design

Thirty rats were involved in this study and were divided into three groups. The first group (*N* = 6) served as a control. The second group involved 12 rats with experimentally induced hypothyroidism. Hypothyroidism in the rats was induced by receiving (0.05% w/v) propylthiouracil which was mixed with drinking water for 1 month.^40^ The hypothyroidism‐induced rats were divided into two subgroups of six rats each. The first subgroup served as a positive hypothyroid control group and the second subgroup received a daily dose of eplerenone (100 mg/kg) by oral gavage for 14 days.^41^

The third group consisted of 12 rats with experimentally induced hyperthyroidism. Hyperthyroidism was induced by receiving L‐thyroxine (0.0012% w/v) for 30 days.^42^ Rats in the third group were subdivided into two subgroups of six each. The first subgroup served as a positive hyperthyroid control group, and the second subgroup received daily doses of eplerenone (100 mg/kg) by oral gavage. Measuring blood pressure, heart rate, Glomerular filtration rate, urine flow, Na^+^ excretion rate, amount of Na^+^ filtered and percentage of Na^+^ reabsorbed from the renal tubules were measured for all groups at the first day, 30th day (after induction of hyperthyroidism and hypothyroidism) and 44th day, 4 h after taking the final dose of eplerenone.

The systolic blood pressure and heart rate were measured by noninvasive technology, putting rats into suitable holders and keeping rats calm and with minimal unexpected noises at morning times. Glomerular filtration rate was determined by measuring renal clearance of endogenous creatinine.^43^

$$\text{Creatinine}\;\text{clearance}=\frac{\text{Urine}\;\text{creatinine}\times \text{urine}\;\text{volume}}{\text{serum}\;\text{creatinine}\times \text{time}\;\text{for}\;\text{urine}\;\text{collection}}$$

At the end of the experiment, the rats were anaesthetized with intraperitoneal injection Ketamine 45 mg/kg and Xylazine 21 mg/kg, blood was withdrawn by using the cardiac puncture technique, serum samples were kept in special test tubes for biochemical assays of renin, angiotensin I, angiotensin II, aldosterone and TSH by ELISA, Sandwich‐ELISA principle was used to estimate serum levels of the parameters, and the micro‐ELISA plate provided in this kit has been precoated with an antibody specific to rat’s protein. Standards or samples were added to the micro‐ELISA plate wells and combined with the specific antibody. Then, a biotinylated detection antibody specific for specific parameter and Avidin‐Horseradish Peroxidase (HRP) conjugate were added successively to each microplate well and incubated. Free components were washed away. The substrate solution was added to each well. Only those wells that contain rat’s protein, biotinylated detection antibody and Avidin HRP conjugate appeared blue in colour. The enzyme‐substrate reaction was terminated by the addition of stop solution and the colour turned yellow. The optical density (OD) was measured spectrophotometrically at a special wavelength of 450 nm ± 2 nm. The OD value was proportional to the concentration of rat’s protein.

Average of the duplicate readings for each standard and samples was taken, and then, the average zero standard optical density was subtracted. four‐parameter logistic curve on log–log graph paper was plotted, with standard concentration on the x‐axis and OD values on the y‐axis. The actual concentration is the calculated concentration multiplied by the dilution factor.

Cobas was used to determine the serum levels of T3, T4, urea, creatinine, Serum Na^+^ and K^+^ were estimated by 9180 electrolyte analyser. A flame photometer was used to detect urine Na^+^ and K^+^ concentration. Urine was collected using a specially designed cylindrical cage, to which a funnel was attached to deliver the urine into the collecting container. Urine volume for each sample was measured that was used for estimating GFR and excretion rate, and then, it was tested freshly for sodium, potassium, and creatinine.

### Statistical analysis

Data were analysed statistically using Statistical Package for Social Sciences (SPSS) version 23.0 for Windows. All the data were expressed as mean ± SE. Comparisons between groups were done using Duncan’s test and the student’s *t*‐test. A *P*‐value of 0.05 or less was considered statistically significant.

## Results

### Induction of hypothyroidism and hyperthyroidism in rats

The results in Table 1 reveal that T3, T4 and TSH serum levels were changed after the administration of propylthiouracil (0.05% w/v) and L‐thyroxine (0.0012% w/v) in the drinking water for one month, respectively. In rats with hypothyroidism, T3 and T4 were significantly reduced from 1.7383 nmol/l to 1.0268 nmol/l and from 78.88 nmol/l to 17.5217 nmol/l, respectively, while TSH was significantly increased from 6.08 ng/ml to 19.5483 ng/ml. In hyperthyroid group, after oral administration of L‐thyroxin the T4 level was increased significantly to 295.6167 nmol/l, while TSH was significantly decreased to 1.2125 ng/ml.

**Table 1:** Thyroid function tests in normal and induced hypothyroidism and hyperthyroidism

Parameter	Control	Hypothyroidism	Hyperthyroidism
T3 (nmol/l)	(1.7383 ± 0.40)	(1.0268 ± 0.081)*	(2.3333 ± 0.29)
T4 (nmol/l)	(78.88 ± 16.55)	(17.5217 ± 4.37)*	(295.6167 ± 16.15)**
TSH (ng/ml)	(6.08 ± 0.89)	(19.5483 ± 4.26)*	(1.2125 ± 0.25)**

Values are expressed as mean ± SEM.

* Compared with the control group *P‐*value less than 0.05.

** Compared with the control group *P‐*value less than 0.001.

### Effect of eplerenone on the RAAS in rats with induced thyroid disorder

**Figure 1:**
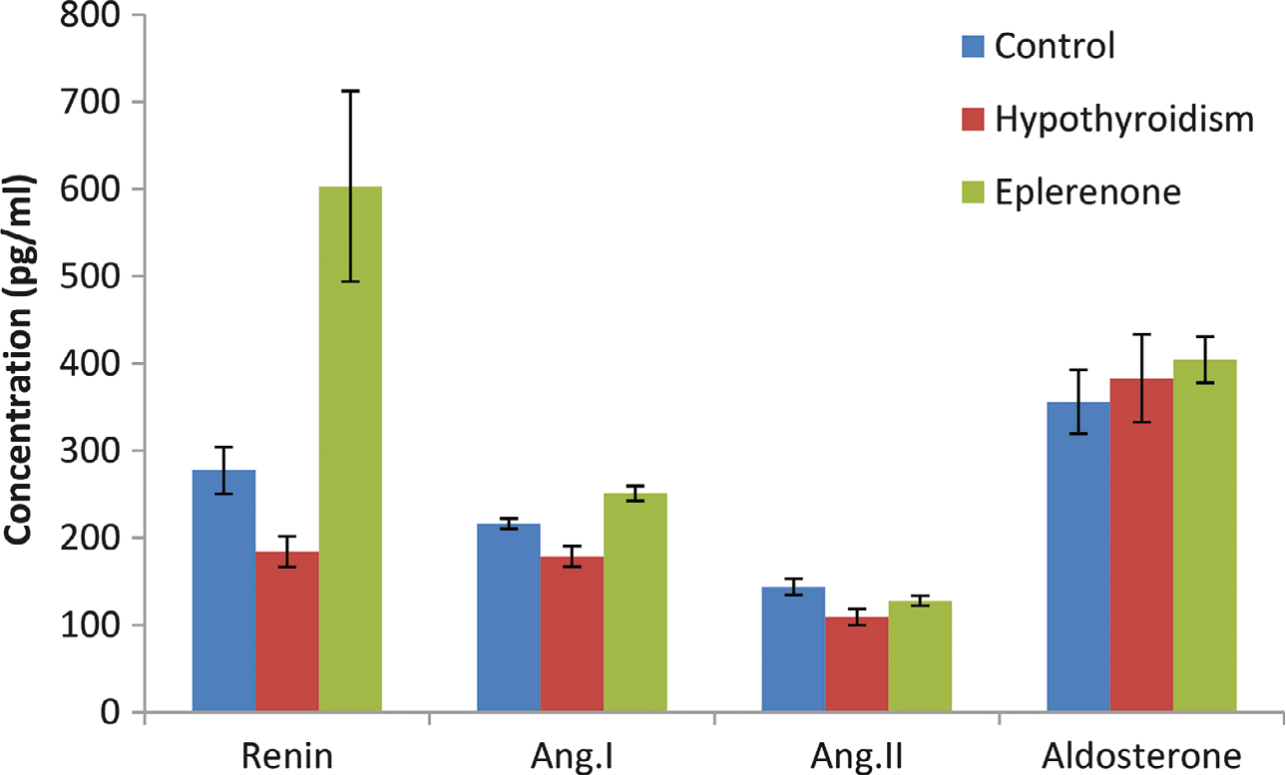
Effects of eplerenone on RAAS in rats with hypothyroidism [Colour figure can be viewed at http://wileyonlinelibrary.com]

**Figure 2:**
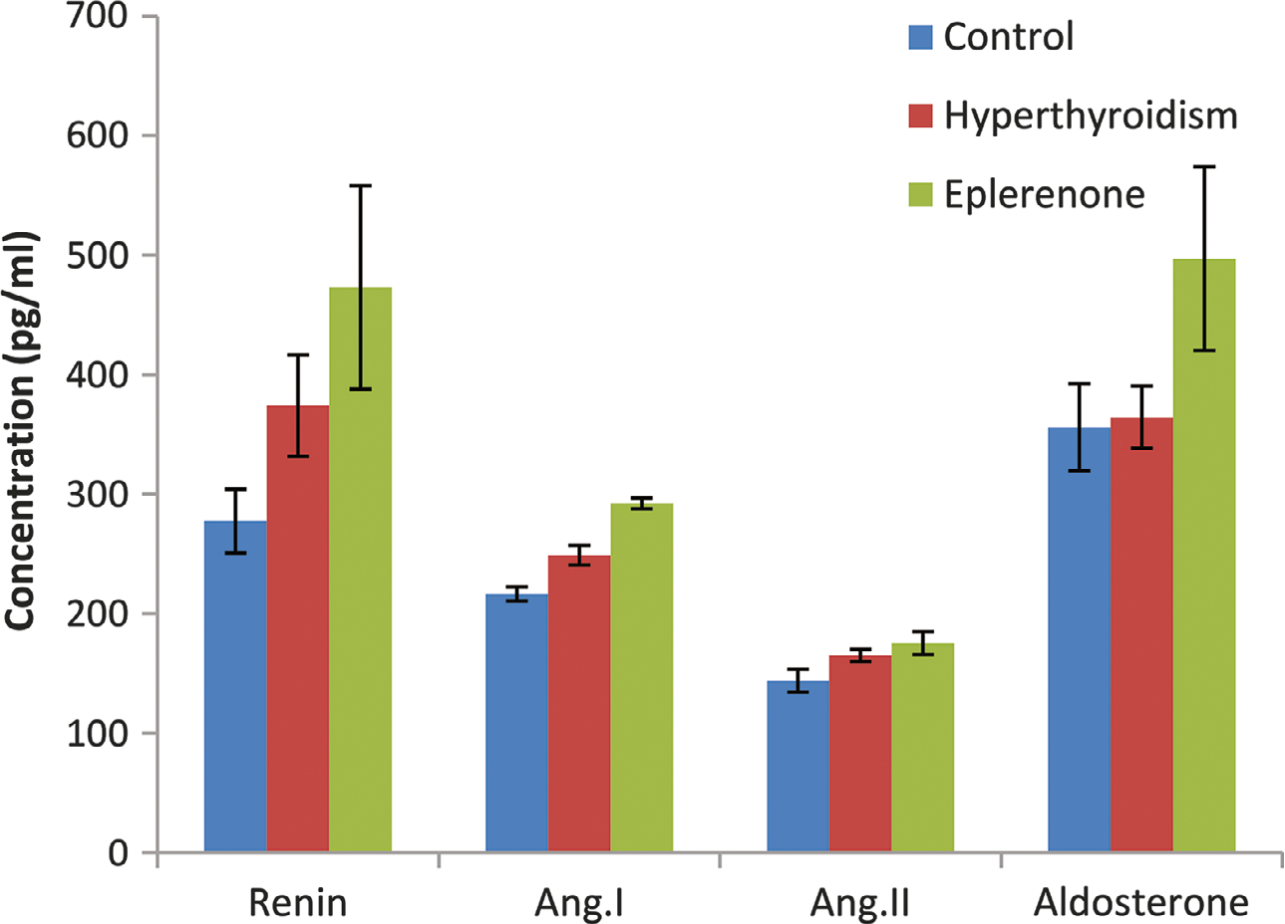
Effects of eplerenone on RAAS in rats with hyperthyroidism [Colour figure can be viewed at http://wileyonlinelibrary.com]

The results presented in Tables 2 and 3 and Figures 1 and 2 indicate the effects of eplerenone on renin, angiotensin I, *angiotensin* II and aldosterone in rats with experimentally induced hypothyroidism and hyperthyroidism. Eplerenone significantly increased the serum renin of hypothyroid rats from 184.0938 pg/ml to 603.3104 pg/ml. Angiotensin I and II were significantly reduced after the induction of hypothyroidism, while after using eplerenone for 14 days in hypothyroid rats angiotensin I significantly increased from 178.6618 pg/ml to 250.8897 pg/ml. The aldosterone level did not change significantly after the induction of hypothyroidism and the administration of eplerenone.

**Table 2:** Effects of eplerenone on RAAS in rats with hypothyroidism

Parameter	Control	Hypothyroidism	Eplerenone
Renin (pg/ml)	277.4552 ± 26.82 a	184.0938 ± 17.52 a	603.3104 ± 109.22 b
Ang. I (pg/ml)	216.3022 ± 5.94 b	178.6618 ± 11.7 a	250.8897 ± 8.46 c
Ang. II (pg/ml)	143.8121 ± 9.44 a	109.1591 ± 9.04 b	127.8831 ± 5.64 ab
Aldosterone (pg/ml)	356.0719 ± 36.57 a	382.9868 ± 50.50 a	404.4772 ± 26.43 a

Values are expressed as mean ± SEM. Different letters indicates significances.

**Table 3:** Effect of eplerenone on RAAS in rats with hyperthyroidism

Parameter	Control	Hyperthyroidism	Eplerenone
Renin (pg/ml)	277.4552 ± 26.82 a	373.9904 ± 42.41 ab	472.8766 ± 85.17 b
Ang. I (pg/ml)	216.3022 ± 5.94 a	248.8431 ± 8.12 b	292.2269 ± 4.52 c
Ang. II (pg/ml)	143.8121 ± 9.44 a	164.9603 ± 4.97 ab	175.0053 ± 9.60 b
Aldosterone (pg/ml)	356.0719 ± 36.57 a	364.2338 ± 25.99 a	497.0254 ± 76.99 b

Values are expressed as mean ± SEM.

In hyperthyroid rats, it was observed that angiotensin I was increased significantly, as indicated in Table 3. The induction of hyperthyroidism had no detectable effect on serum aldosterone in rats. Meanwhile angiotensin I and aldosterone were significantly increased in the eplerenone‐treated group when compared with hyperthyroid rats.

### Effect of eplerenone on renal functions, serum urea and serum creatinine in rats with hypothyroidism

The results presented in Table 4 indicate the effects of eplerenone on renal functions, serum urea and serum creatinine in experimentally induced hypothyroidism rats, as it can be seen that the urine flow and sodium excretion rate were increased significantly after the oral administration of eplerenone for two weeks, to 1.41521 ml/hr/kg and 0.3172 ml/hr/kg, respectively, when compared with both the control and hypothyroid rats. The GFR and amount of sodium filtered were increased significantly after administration of eplerenone. The percentage of reabsorbed sodium was decreased significantly after the administration of eplerenone to 99% in hypothyroid rats. Hypothyroidism had no significant effect on the total solute excretion rate of rats; however, eplerenone could significantly increase the total solute excretion rate. Serum urea was significantly decreased in eplerenone‐treated rats when compared with both normal and hypothyroid rats.

**Table 4:** Effect of eplerenone on renal functions, serum urea and serum creatinine in rats with hypothyroidism

Parameter	Control	Hypothyroidism	Eplerenone
Urine flow (ml/h/kg)	0.7718 ± 0.09 a	0.6189 ± 0.06 a	1.41521 ± 0.18 b
Na exc. Rate (mmole/h/kg)	0.1493 ± 0.02 ab	0.0534 ± 0.08 a	0.3172 ± 0.11 b
GFR (mmol/h/kg)	165.56 ± 25.13 ab	112.31 ± 13.26 a	236.49 ± 47.62 b
Amount of Na filtered (mmol/h/kg)	22.7123 ± 3.56 ab	16.4734 ± 1.88 a	30.8122 ± 6.11 b
Percentage of Na reabsorbed	99.2567 ± 0.18 ab	99.6433 ± 0.08 b	99 ± 0.17 a
Total solute excreted (meq/min)	0.5715 ± 0.04 a	0.4168 ± 0.05 a	1.1613 ± 0.15 B
S. urea (mg/dl)	43.833 ± 2.13 a	44.967 ± 1.34 a	36.75 ± 1.26 b
S. creatinine (mg/dl)	0.55 ± 0.034 a	0.6133 ± 0.054 a	0.5350 ± 0.026 a

Values are expressed as mean ± SEM.

### Effect of eplerenone on renal functions, serum urea and serum creatinine in rats with hyperthyroidism

The results presented in Table 5 reveal that eplerenone could significantly increase urine flow, sodium excretion rate, GFR, amount of sodium filtered and total solute excreted of hyperthyroid rats. In contrast to the above results, the percentage of sodium reabsorbed was significantly reduced by the daily use of 100 mg/kg of eplerenone.

**Table 5:** Effect of eplerenone on renal functions, serum urea and serum creatinine in rats with hyperthyroidism

Parameter	Control	Hyperthyroidism	Eplerenone
Urine flow(ml/h/kg)	0.7718 ± 0.09 a	1.3448 ± 0.24 a	3.6098 ± 1.00 b
Na exc. Rate (mmol/h/kg)	0.1493 ± 0.02 a	0.1821 ± 0.03 a	0.9907 ± 0.29 b
GFR(mmol/h/kg)	165.5688 ± 25.13 a	226.0935 ± 22.99 a	460.2065 ± 78.01 b
Amount of Na filtered (mmol/h/kg)	22.7123 ± 3.56 a	31.4493 ± 3.40 a	64.7003 ± 11.05 b
Percentage of Na reabsorbed	99.2567 ± 0.183 a	99.3983 ± 0.130 a	98.6180 ± 0.287 b
Total solute excreted (meq/min)	0.5715 ± 0.048 a	0.6970 ± 0.157 a	1.3843 ± 0.164 b
S. urea (mg/dl)	43.833 ± 2.13 a	44.5 ± 3.74 a	31.76 ± 1.63 b
S. creatinine (mg/dl)	0.55 ± 0.034 a	0.45 ± 0.022 b	0.392 ± 0.016 b

Values are expressed as mean ± SEM.

Serum creatinine in hyperthyroid and eplerenone‐treated rats was slightly but significantly lower than that of normal rats. No detectable change was observed in serum urea in experimentally induced hyperthyroid rats when compared to normal rats; however, eplerenone significantly reduced the serum urea from 44.5 to 31.76 in hyperthyroid rats.

### Effect of eplerenone on systolic blood pressure and heart rate in rats with hypothyroidism

The systolic blood pressure in hypothyroid rats was not significantly changed when compared with normal rats, while eplerenone significantly reduced it (Table 6). The results presented in Table 6 indicate that the heart rate in hypothyroid and eplerenone‐treated rats was significantly lower than that of normal rats.

**Table 6:** Effect of eplerenone on systolic blood pressure and heart rate in rats with hypothyroidism

Parameter	Control	Hypothyroidism	Eplerenone
Systolic (mmHg)	112 ± 1.43 a	107.66 ± 1.14 a	93.41 ± 3.73 b
Heart rate (beat/min.)	381 ± 15 a	308 ± 3 b	306 ± 2.34 b

Values are expressed as mean ± SEM.

### Effect of eplerenone on systolic blood pressure and heart rate in rats with hyperthyroidism

The systolic blood pressure was significantly increased in hyperthyroid rats when compared with normal rats (Table 7); however, no significant changes were observed in the heart rates of hyperthyroid rats when compared with normal rats. The systolic blood pressure of hyperthyroid rats which had received eplerenone for two weeks was significantly reduced, as presented in Table 7.

**Table 7:** Effect of eplerenone on systolic blood pressure and heart rate in rats with hyperthyroidism

Parameter	Control	Hyperthyroidism	Eplerenone
Systolic (mmHg)	115.2 ± 2.55 a	127 ± 3.77 b	104.2 ± 3.39 a
Heart rate (beat/min.)	356 ± 19 a	390 ± 10 a	366 ± 7 a

Values are expressed as mean ± SEM.

## Discussion

It is clear from Table 1 that T3, T4 and TSH serum levels were markedly changed after the administration of propylthiouracil (0.05% w/v) and L‐thyroxine (0.0012% w/v) for 30 days to rats in the hypothyroid and hyperthyroid groups, respectively. T3 and T4 serum levels were decreased significantly after receiving propylthiouracil and induced a significant change in the serum TSH level when compared with the control group.^40^ In the hyperthyroid group, T4 was increased significantly, while TSH decreased significantly, which is another indicator for the successful induction of hyperthyroidism, in accordance with a previous study.^42^

In this study, the serum level of renin was decreased in hypothyroid rats; this change most probably is due to the reduction of the metabolic rate and dysfunction of juxtaglomerular cells, which is the site of renin release;^44^, ^45^ in addition, the hypothyroidism state decreases the sensitivity of β‐adrenergic receptors, which is another stimulation mechanism for renin release.^33^ After oral administration of eplerenone for 14 days, the serum renin concentration increased significantly. This change might be due to hypotension triggered by the stimulation of intrarenal baroreceptors, which consequently stimulates renin release as a compensatory mechanism.^46^ The serum levels of angiotensin I and II were decreased significantly in rats with hypothyroidism, possibly because of a reduction of the serum renin concentration caused by thyroid dysfunction.^47^, ^48^

The serum levels of angiotensin I and II were increased in hypothyroid rats that had received eplerenone. These changes might be due to an increase in the serum renin concentration, as a result of a compensatory mechanism in response to hypotension caused by the aldosterone receptor blocker.

In this study, hypothyroid disorder had no significant effect on the aldosterone concentration in rats. This result is in agreement with a previous cross‐sectional study by Asmah, B. J.* et al.*, who concluded that the basal plasma aldosterone concentration was not significantly different in either hypothyroidism or hyperthyroidism.^49^ Giving eplerenone to hypothyroid rats did not significantly change the aldosterone concentration, because the synthesis and release of aldosterone in the adrenal cortex are not affected by an aldosterone receptor blocker such as eplerenone.

The serum levels of renin, angiotensin I and II were increased after inducing hyperthyroidism by L‐thyroxine. This change could contribute to an increase in the sympathetic outflow as a result of increased thyroid hormone, since the stimulation of β receptors by released adrenalin enhances the release of renin, consequently, angiotensin I and II concentrations were increased.^35^, ^36^, ^37^

After oral administration of eplerenone to the hyperthyroid rats, the serum concentrations of renin, angiotensin I and II were increased; this increase might be related to a compensatory mechanism induced by a reduction in blood pressure mediated by intrarenal baroreceptors. However, a reduction of the sodium concentration in the renal tubule lumen cannot be excluded.

In the current study, eplerenone significantly increased the serum aldosterone in rats with hyperthyroidism, since blockage of aldosterone receptors by eplerenone can lead to an increase of free aldosterone as a compensatory mechanism to overcome blocking of aldosterone receptors antagonized by eplerenone.^46^

The urine flow, sodium excretion rate, GFR, amount of sodium filtered and total solute excreted were decreased non‐significantly after inducing hypothyroidism by the oral administration of propylthiouracil; these changes are possibly due to a reduction of renal blood flow through a reduction of cardiac contractility and heart rate caused by a lowered level of thyroid hormone,^50^ in addition to a decrease in the sensitivity of the β‐adrenergic receptors, which in turn reduces the renin release and angiotensin II concentration.^33^

It is well known that aldosterone increases the expression of sodium channels and sodium–potassium ATPase in the late distal tubule and collecting ducts. This expression leads to absorption of sodium from the lumen and allows water absorption. This directly results in an increase in osmolality within the blood, causing water to flow down its concentration gradient.^51^ Eplerenone as an aldosterone receptor blocker leads to a reduction in urine flow, sodium excretion rate, GFR, amount of sodium filtered and total solute excreted in hypothyroid rats.

After oral administration of eplerenone, the percentage of sodium reabsorbed slightly but significantly decreased when compared with hypothyroid rats, which may be due to a reduction of the activity of the basolateral Na^+^/K^+^ ATPase and apical Na^+^–H^+^ exchanger induced by eplerenone.^52^, ^53^

After inducing hyperthyroidism by the oral administration of L‐thyroxine, urine flow, sodium excretion, GFR, amount of sodium reabsorbed and total solute excreted were increased, but the changes were not significant when compared with the control group. These changes related to many reasons: first, increasing the cardiac output, and second, the renal blood flow due to the effect of the thyroid hormone.^45^ At the same time, increasing the activity of the RAAS also played a role in these changes.^33^

After treatment of the rats with hyperthyroidism with eplerenone, the level of urine flow, sodium excretion, GFR, amount of sodium reabsorbed and total solute excreted increased, which is due to the blockage of the aldosterone receptor and decrease in the activity of the sodium channels and Na^+^/K^+^ ATPase.^51^

The percentage of sodium reabsorbed increased after inducing hyperthyroidism, but the change was not significant compared with the control group, while after the oral administration of eplerenone the level decreased significantly, which is due to the effect of eplerenone on the aldosterone receptor.^52^, ^53^

The serum creatinine level increased in rats with induced hypothyroidism but the change was not significant, while in the hyperthyroid group, the level was significantly reduced; these changes are in agreement with another study.^54^ After the oral administration of eplerenone to hypothyroid rats, the level decreased but the change was not significant. The serum creatinine was significantly decreased in hyperthyroid rats treated with eplerenone compared with euthyroid rats, probably due to the increased GFR as a result of eplerenone.

In hypothyroid and hyperthyroid groups the serum urea did not change significantly; while eplerenone significantly decreased the serum urea, possibly due to an increase in GFR induced by eplerenone.

The systolic blood pressure in hypothyroid rats did not change significantly when compared with euthyroid rats, while it was increased significantly in hyperthyroid rats; these changes are probably due to sympathetic activity which decreased in hypothyroidism and increased in hyperthyroidism.^35^

Hypothyroid and hyperthyroid rats receiving eplerenone exhibited a significant decrease in systolic blood pressure, possibly due to the diuretic effect of eplerenone.

Compared with the normal rats, the heart rate in hypothyroid rats was significantly decreased, but it was non‐significantly increased in hyperthyroid rats; these changes are related to the sympathetic activities in thyroid disorders.^35^ At the same time, eplerenone caused no significant change in heart rate either in the hypothyroid or hyperthyroid groups.

## Conclusion

Eplerenone significantly increased serum renin and angiotensin I in hypothyroid rats and aldosterone and angiotensin I in hyperthyroid rats, when compared with the positive controls. Aldosterone in hypothyroid rats was not changed by eplerenone. In hypothyroid and hyperthyroid rats, eplerenone significantly increased the urine flow, sodium excretion rate, GFR, amount of sodium filtered, percentage of sodium reabsorbed, total solute excreted and serum urea, while there was no significant change in serum creatinine. There was also a significant change in systolic blood pressure, while there was no significant change in heart rate in either the hypothyroid or hyperthyroid rats.

## Declarations

### Conflict of interest

The authors report no conflicts of interest in this work.
